# Wound healing, calcium signaling, and other novel pathways are associated with the formation of butterfly eyespots

**DOI:** 10.1186/s12864-017-4175-7

**Published:** 2017-10-16

**Authors:** Nesibe Özsu, Antónia Monteiro

**Affiliations:** 1 Department of Biological Sciences, National University of Singapore, Singapore, 117543 Singapore; 2 Yale-NUS College, Singapore, 138614 Singapore

**Keywords:** Novel traits, Co-option, Transcriptome, Gene expression, *Toll*, *T-box 6*

## Abstract

**Background:**

One hypothesis surrounding the origin of novel traits is that they originate from the co-option of pre-existing genes or larger gene regulatory networks into novel developmental contexts. Insights into a trait’s evolutionary origins can, thus, be gained via identification of the genes underlying trait development, and exploring whether those genes also function in other developmental contexts. Here we investigate the set of genes associated with the development of eyespot color patterns, a trait that originated once within the Nymphalid family of butterflies. Although several genes associated with eyespot development have been identified, the eyespot gene regulatory network remains largely unknown.

**Results:**

In this study, next-generation sequencing and transcriptome analyses were used to identify a large set of genes associated with eyespot development of *Bicyclus anynana* butterflies, at 3-6 h after pupation, prior to the differentiation of the color rings. Eyespot-associated genes were identified by comparing the transcriptomes of homologous micro-dissected wing tissues that either develop or do not develop eyespots in wild-type and a mutant line of butterflies, Spotty, with extra eyespots. Overall, 186 genes were significantly up and down-regulated in wing tissues that develop eyespots compared to wing tissues that do not. Many of the differentially expressed genes have yet to be annotated. New signaling pathways, including the Toll, Fibroblast Growth Factor (FGF), extracellular signal–regulated kinase (ERK) and/or Jun N-terminal kinase (JNK) signaling pathways are associated for the first time with eyespot development. In addition, several genes involved in wound healing and calcium signaling were also found to be associated with eyespots.

**Conclusions:**

Overall, this study provides the identity of many new genes and signaling pathways associated with eyespots, and suggests that the ancient wound healing gene regulatory network may have been co-opted to cells at the center of the pattern to aid in eyespot origins. New transcription factors that may be providing different identities to distinct wing sectors, and genes with sexually dimorphic expression in the eyespots were also identified.

**Electronic supplementary material:**

The online version of this article (10.1186/s12864-017-4175-7) contains supplementary material, which is available to authorized users.

## Background

The evolution of novel traits and of their underlying gene regulatory networks remains a hot topic in the field in evolutionary biology. Some novel traits appear to have arisen via the qualitative modification of pre-existing traits with more primitive features [1, 2]. Examples of such novel traits include angiosperm flowers derived via the modification of the leaves into sepals and petals [3], the novel mammary gland derived from a skin gland that started secreting novel compounds [4], or of butterfly eyespots on the wing with concentric rings of color from simpler spots [5, 6]. When such evolutionary transitions take place, a fundamental question that follows pertains to the type and nature of the molecular modifications that took place to lead to the origin of the novel trait.

Proposed mechanisms for the origin of novel traits include the fusion of distinct cell types, as in the case of the insect wing [7–9], or the recruitment of pre-existing gene regulatory networks into novel developmental contexts [10–12]. In these latter cases, cells from tissues that build the novel trait generally display novel gene expression profiles due to the activation of new gene regulatory networks in those cells. Comparing the gene expression profile of these cells to those of cells serving distinct functions in the body can help identify ancestral and pre-existent gene regulatory networks that were recruited to help build the novel trait [13]. This type of research recently pointed to a novel adaptive trait “beetle horns” having likely been co-opted from the limb gene regulatory network [14], and to a novel lobe in the adult genitalia of *Drosophila* originating from the recruitment of a gene regulatory network involved in differentiating larval breathing spiracles earlier in development [11]. Hence, identifying the developmental origin of novel traits can begin with the identification of the underlying genes involved in building the novel trait.

In this study, we are interested in investigating the nature of the gene regulatory network underlying eyespot development. Eyespots are novel traits on the wings of butterflies that serve adaptive roles in both predator avoidance and sexual signaling [15–22]. Previous research suggested that nymphalid butterfly eyespots originated once via the co-option of pre-existing gene networks [5, 6, 23]. Network co-option was suggested because the expression of four out of five genes examined in association with eyespot development in 23 species of nymphalids and outgroups was inferred to have originated concurrently with the origin of eyespots [6]. These data suggest that these genes may not have been recruited gradually and individually to help build the novel trait, but appeared associated with the trait in a more abrupt way via the co-option of a gene regulatory network wherein the genes were already connected to each other. Previous work, however, had already hinted at co-option events being associated with eyespot origins by the type of gene being discovered in association with eyespots. For instance, the first gene ever to be visualized in eyespots, *Distal-less*, pointed at a possible limb network co-option event [24]. Later, the hedgehog regulatory circuit involved in patterning the anterior-posterior axis of insect wings was also proposed [25]. This was followed by the wound healing gene regulatory network [26], and a regulatory circuit involved in wing margin development [27]. For a clearer understanding of which, if any, of these circuits and/or networks may have been co-opted to aid in eyespot origins, as well as the discovery of new genes or networks that may have aided in eyespot origins, it is important to examine the complete set of genes involved in eyespot development.

Eyespot development in nymphalid butterflies has been studied since 1978 [28], but so far only 12 genes have been discovered associated with eyespots in two nymphalid model species, *Junonia coenia* and *Bicyclus anynana* (reviewed in Monteiro 2015) mostly developed against fly proteins. Thanks to innovations in gene expression profiling, such as transcriptome analysis on next-generation sequencing platforms, it is now possible to compare total gene expression across different cell types or tissues [29], and potentially identify the complete set of genes associated with eyespot development.

**Fig. 1:**
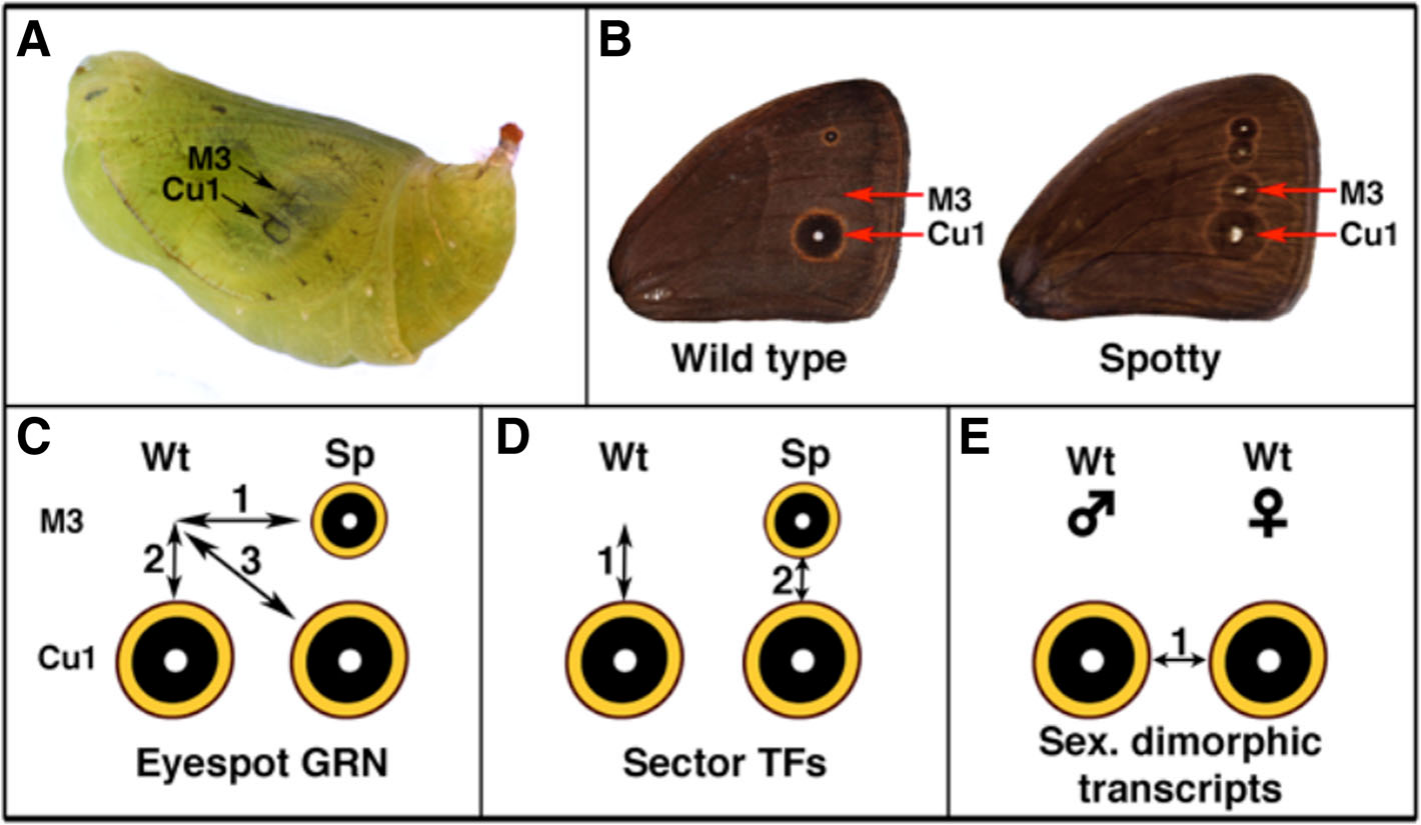
Experimental approach to identify eyespot associated genes, sector-specific transcription factors (TFs), and sex-biased eyespot gene expression. **a** Micro-dissected tissues were collected from two wing sectors, M3 and Cu1 during the early pupal stage. **b** The Spotty mutation adds two additional eyespots to the wing, one in sector M3. **c** Genes associated with eyespot development were identified as the set of genes that were differentially expressed between wing sectors that do not develop eyespots versus wing sectors that develop eyespots across three comparisons between homologous and non-homologous wing sectors of wild-type (Wt) and Spotty (Sp) wings. **d** Wing sector specific TFs were identified as the set of common differentially expressed genes between wing sectors M3 and Cu1 in both Wt and Sp wings. **e** Sexually dimorphic transcripts were identified as differentially expressed genes between Cu1 male (Wt) and Cu1 female (Wt) wing sectors

In this study, we used next-generation sequencing and transciptome analyses to identify the set of genes associated with eyespot development in the early pupal stage of *B. anynana*. This stage corresponds to an important signaling stage of eyespot development where cells at the center of the pattern signal to surrounding cells to differentiate the color rings [30–32]. In addition, this stage was chosen because of practical considerations. The fragile dorsal wing surface epidermis remains attached to the sturdier overlaying cuticle for a few hours after pupation, and this facilitates the dissection of very small pieces of epidermis containing the eyespot central cells, the pattern organizers. We used micro-dissections of wing epidermis from wild-type and from an eyespot mutant of *B. anynana*, Spotty, with additional eyespots on the wing, to compare the transcriptome of homologous wing sectors with and without eyespots (Fig. 1a). In addition, this approach also allowed us to identify transcription factors differentially expressed between adjacent wing sectors of the forewing, and identify differential expression of genes in male and female eyespots.

## Results

### Transcriptome analysis

Around 400 million paired end reads (~100 bp) were generated from 15 different mRNA libraries. These libraries consisted of three biological replicates of micro-dissected tissue from two distinct wing sectors in males (Cu1 and M3) of wild-type and Spotty individuals, and three biological replicates from a single wing sector in wild-type females (Cu1; Fig. 1). A total of 135,491 contigs were assembled with a N50 value of 1061 bp. The length of the longest contig was 11,724 bp. Around 95% of reads from each library were mapped back to the transcriptome to estimate transcript abundance.

### Clustering analysis

A clustering analysis that compared transcripts across all samples (15 libraries) was performed to check data quality. The analysis revealed that biological replicates and samples from the same type of tissue generally clustered together (Additional file 1: Figure S1). Differentially expressed genes were identified across the 15 libraries using a false discovery rate (FDR) *p*-value of <0.001 and at least a 2-fold change in gene expression. First, wild-type and Spotty mutant samples were clearly separated from each other indicating many genes were differentially expressed between these tissue samples. Second, within wild-type butterfly tissue, female samples were separated from male samples. Third, M3 and Cu1 wing sectors of wild-type male tissues were separated from each other. Lastly, all samples from Spotty wings, all containing eyespots, clustered together, but one biological replicate of the Spotty Cu1 wing sector clustered closest to tissue from the Spotty M3 wing sector. This could be due to a small number of differentially expressed genes between Cu1 and M3 wing sectors in Spotty wings (see following).

### The *Bicyclus anynana* eyespot-associated differentially-expressed transcripts at early pupation

Genes associated with eyespot development were identified as the common set of differentially expressed genes between wing sectors that do not develop eyespots versus wing sectors that develop eyespots (Table 1). Presumably up-regulated genes represent novel gene expression patterns due to genetic co-option events associated with eyespot development, whereas down-regulated genes represent the downstream effects of these co-options on downstream targets, previously expressed at some basal level in tissues without eyespots. We calculated three sets of differentially expressed genes: between homologous M3 sectors in Wt and Spotty wings; between M3 and Cu1 sectors in Wt wings; and between M3 sectors in Wt wings and Cu1 sectors in Spotty wings (Fig. 1c). These analyses revealed the common set of transcripts that were significantly up- and down-regulated in a similar way across the three comparisons.

**Table 1:** Total number of differentially expressed transcripts between pairs of wing sectors that led to the identification of eyespot-specific, sector-specific, and sex-specific transcripts

Objective	Comparisons	Total differentially expressed transcripts	Up-regulated transcripts	Down-regulated transcripts	Common up-regulated transcripts	Common down-regulated transcripts
Genes associated with eyespot development	Sp M3 vs. Wt M3	4056	2088	1968	132	54
	Wt Cu1 vs. Wt M3	1368	792	576		
	Sp Cu1 vs. Wt M3	2197	1066	1131		
Sector specific transcripts	Wt M3 vs. Wt Cu1	1368	576	792	23	29
	Sp M3 vs. Sp Cu1	1169	628	541		
Differentially expressed transcripts between sexes	Male Cu1 vs. Female Cu1	2785	1714	1071	1714	1071

A total of 186 transcripts were identified as being associated with eyespot development (Fig. 1c). From these transcripts, 132 were up-regulated and 54 transcripts were down-regulated in the tissues that develop eyespots versus the tissues that do not (Table 1). The data was processed as follows: First, 4056 transcripts were found differentially expressed in M3 wing sectors between Spotty and wild-type (Wt) wings (Fig. 1c). From these transcripts, 2088 were up-regulated and 1968 were down-regulated in the M3 sector with eyespots (in Spotty). Second, 1368 transcripts were differentially expressed between Cu1 and M3 wing sectors in Wt wings (Fig. 1c). From these, 792 were up-regulated and 576 transcripts were down-regulated in the Cu1 wing sector with eyespots. Third, 2197 transcripts were differentially expressed between Cu1 wing sectors in Spotty and M3 wing sector in Wt (Fig. 1c). Of these, 1066 were up-regulated and 1131 down-regulated in the wing sector with eyespots.

To verify that this set of 186 eyespot genes was stable across the sexes, we compared the transcriptomes of Wt female tissues that develop eyespots (Cu1) with Wt male tissues that do not develop eyespots (M3). Interestingly, we found that 80% of the previously identified 186 eyespot-associated genes were differentially expressed in this comparison as well (Additional file 2: Table S1, the 20% of the genes that were not differentially expressed in this comparison are highlighted in green).

**Fig. 2:**
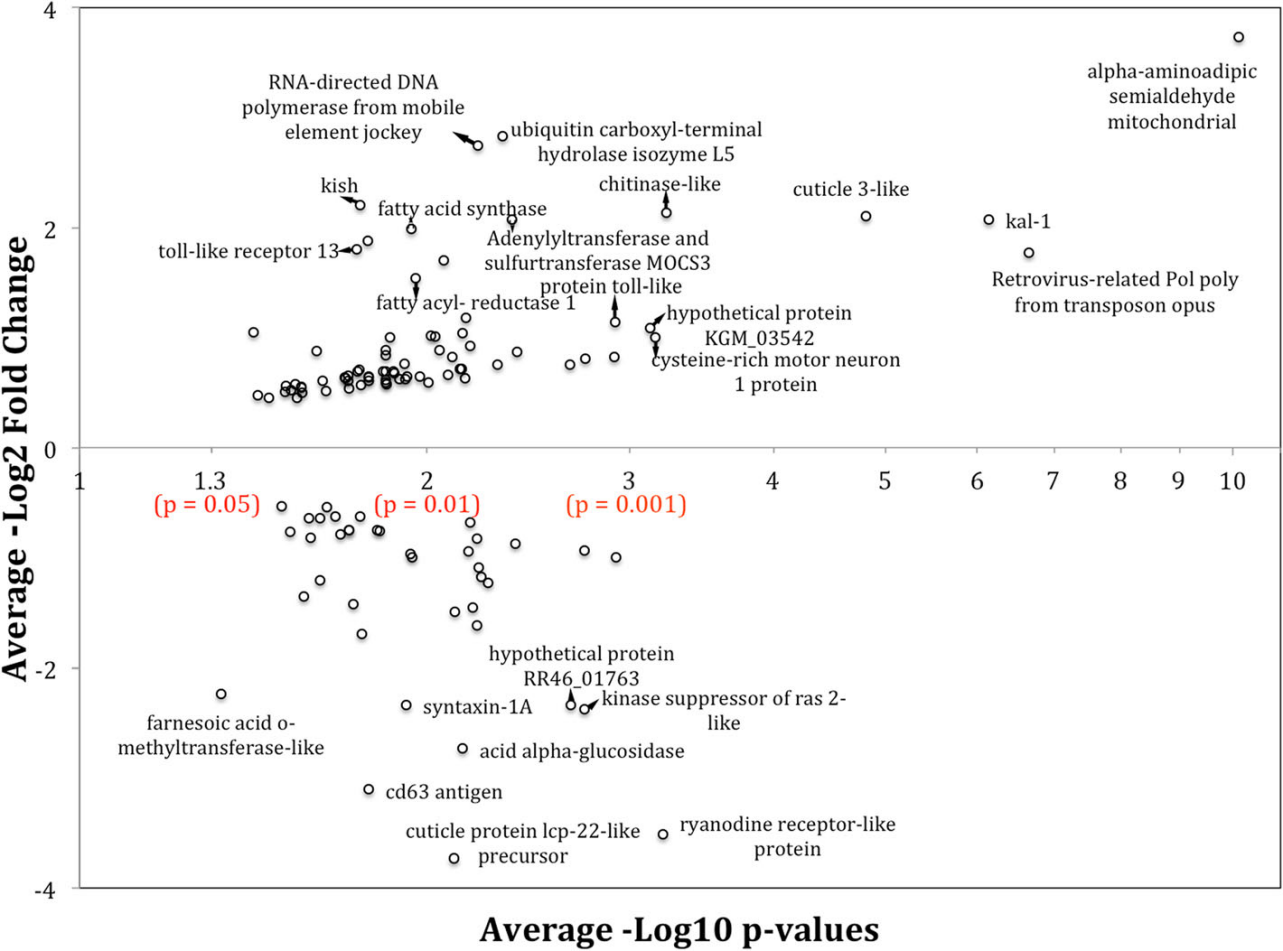
Set of differentially expressed genes between tissues with eyespots and tissues without eyespots observed across three different comparisons. Average fold change in gene expression (log2) is plotted against average negative *p*-values (log10). *P*-values associated with gene expression differences are obtained by taking into account the three biological replicates for each type of tissue sampled. Genes with known annotations are identified in the graph

All differentially expressed genes were blasted and annotated for gene ontology (GO) terms (Additional file 3: Table S14-19). Many of the genes identified (72, 39%) were not previously annotated in the databases and this included some of the most highly up- and down-regulated genes. From the annotated set, some genes showed large fold expression differences that were highly significant (Fig. 2). Examples of such significantly (*p*-value <0.001) up-regulated genes in tissue containing eyespots included *Alpha-aminoadipic semialdehyde mitochondrial*, *kal-1 protein*, *retrovirus-related Pol poly from transposon opus*, *cuticle 3-like*, *cysteine-rich motor neuron 1 protein*, *hypothetical protein KGM_03542* and *chitinase-like protein*. In addition, *RNA-directed DNA polymerase from mobile element jockey*, *ubiquitin carboxyl-terminal hydrolase isozyme*, *kish* and *adenylyltransferase and sulfurtransferase MOCS3* were also four-fold up-regulated in eyespot tissues with a slightly higher *p*-value (*p* < 0.05). Other significantly up-regulated genes in eyespots, at less than two-fold expression level differences, included two Toll-like proteins. Significantly down regulated genes (four-fold) in tissues with eyespots included *ryanodine receptor-like protein*, *larval cuticle protein lcp-22-like precursor*, *acid alpha-glucosidase*, *kinase suppressor of ras 2-like protein*, *hypothetical protein RR46_01763*, *cd63 antigen*, *farnesoic acid o-methyltransferase-like protein* and *syntaxin-1A*.

In order to test whether genes associated with eyespots were enriched for any particular functional category, we performed an enrichment analysis. None of the annotated gene ontology categories were found to be enriched in eyespot tissues (Fisher’s exact test: *p*-value <0.05). This can be due to the fact that only a small portion of the genes had GO terms (Additional file 3: Table S14 and Table S15).

### Candidate genes and candidate networks

**Fig. 3:**
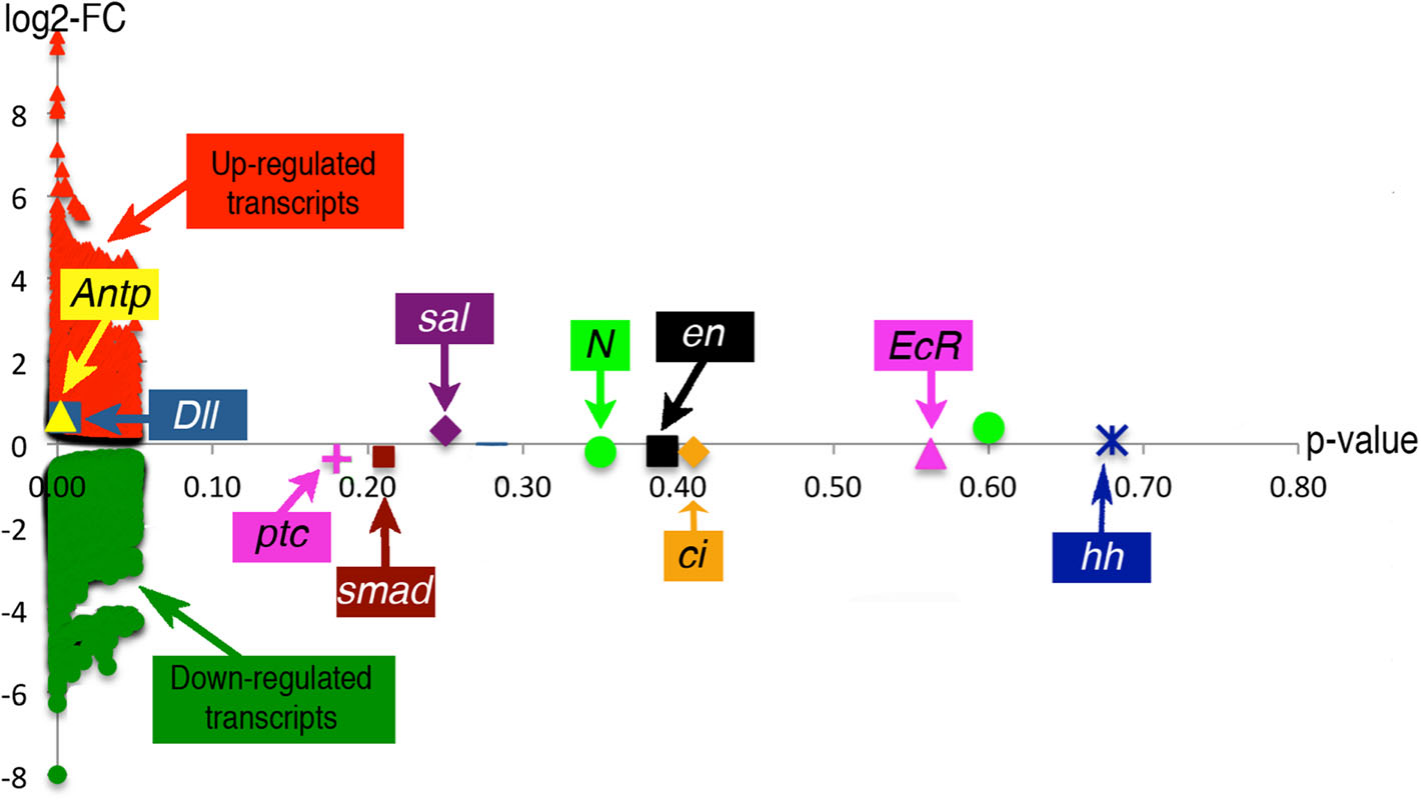
The expression of previously discovered genes associated with butterfly eyespot development. The graph shows the fold change in gene expression (log2) and the p-values of all the differentially expressed transcripts associated with eyespot development in a single comparison across homologous tissues from the M3 sector between wild-type and Spotty. *Antennapedia* (*Antp), Distal-less* (*Dll*), *spalt* (*sal*), *engrailed* (*en*), *cubitus interruptus* (*ci*), *Notch* (*N*), *patched* (*ptc*), *Ecdysone receptor* (*EcR*), *smad, hedgehog* (*hh*)

We examined expression profiles of the genes previously associated with eyespot development in *B. anynana* and in other butterflies in our transcriptome data (Additional file 4: Table S3). With the exception of *Ultrabithorax* and *wingless*, the other ten genes were present in the transcriptome. However, with the exception of *Distal-less* and *Antennapedia,* most of the eyespot-associated genes were not differentially expressed between tissues with eyespots and tissues without eyespots (Fig. 3).

We further manually examined all the annotated genes (114) for a possible known function in the four gene regulatory networks previously proposed to have been co-opted to aid in eyespot origins. Some of the genes have known roles in wound healing (18 genes), limb development (3 genes), anterior-posterior axis establishment and segment polarity (4 genes), and wing margin development (4 genes), whereas most genes have no known role in these developmental contexts (Additional file 5: Table S2).

### Transcription factor differences between M3 and Cu1 wing sectors

During *Drosophila* wing development each sector of the wing expresses a different combination of transcription factors [33], and veins form at boundaries of expression of some of these transcription factors [34, 35]. Butterflies may use similar mechanisms to produce their venation patterns. In addition, these transcription factors due to their presence in only specific sectors of the wing may be used as selector genes to modify the eyespot gene regulatory network in those sectors, i.e., to regulate presence/absence and/or eyespot size in a sector-specific fashion. Hence, Spotty, due to its restricted effect on only two sectors of the wing [36, 37], could be a mutation involving such a selector gene. The mutation could be either in the selector gene itself, or alternatively, in the selector’s downstream targets such as in regulatory DNA flanking eyespot network genes. This latter type of mutation is more likely, as it would have fewer pleiotropic effects such as vein disruptions. Given these assumptions we tried to identify candidate wing sector specific genes by comparing differentially expressed genes between wing sectors M3 and Cu1 in both Wt and Spotty wings, and finding the set of common differentially expressed genes, regardless of the presence and absence of eyespots. A total of 52 transcripts were differentially expressed between wing sectors M3 and Cu1 in *B. anynana*. Of these, 23 transcripts were up-regulated and 29 transcripts were down-regulated in wing sector M3 versus wing sector Cu1 (Table 1). The data was processed as follows: First, we found 576 transcripts up-regulated, and 792 transcripts down-regulated in wing sector M3 relative to Cu1 in Wt butterflies (Fig. 1d). Second, we found 628 transcripts up-regulated and 541 transcripts down-regulated in wing sector M3 relative to Cu1 in the Spotty mutant (Fig. 1d). We then identified the common up, and down-regulated genes between these two comparisons. From this set we identified a single annotated transcription factor, *T-box 6*, expressed at significantly higher levels in the M3 wing sector relative to the Cu1 sector in both Wt and Spotty wings (Wt: *p*-value: 1.51E-14 and logFC: 5.59; Spotty: *p*-value: 8.25E-011 and logFC: 6.89).

### Differentially expressed transcripts between female and male Cu1 eyespots

To analyze differentially expressed transcripts between female and male eyespots, wing tissue samples were compared between Wt Cu1 male and Wt Cu1 female wing sectors (Fig. 1e). We found 2785 differentially expressed transcripts. Of these, 1714 were up-regulated (Additional file 6: Table S12) and 1071 were down-regulated (Additional file 6: Table S13) in males versus females (Table 1).

## Discussion

### Wound healing and immune system genes are associated with eyespot development

A large fraction (16%) of the 114 annotated differentially expressed genes associated with eyespots have a role in wound healing in other systems (Additional file 5: Table S2). Previous work showed that wounding the pupal wing from 6 to 18 h after pupation induced ectopic eyespots around the area of injury [30, 38, 39]. These ectopic eyespots are similar to normal eyespots in that they have a black disc of scales surrounded by a yellow ring, but lack the central white scales [39]. Additionally, the same transcription factors that map to the different color rings are also expressed in ectopic eyespots after wounding [26]. Due to these similarities, Monteiro et al. [26] proposed that eyespot evolution involved the co-option of the wound healing gene regulatory network to aid in eyespot development. Here, the transcriptome analysis lends support to this hypothesis by identifying additional eyespot-associated genes known for their roles in wound repair.

Calcium signaling is an early trigger associated with wound repair, and this signaling pathway appears to be involved in the eyespot gene regulatory network. An increase in intracellular calcium was the proposed trigger in wound healing in worms and humans [40, 41] and wounding a different butterfly, *Junonia orythia*, triggers a rapid calcium wave that spreads from injured areas and leads to temporary Ca^2+^ increases in epidermal cells [42, 43]. One of the up-regulated genes in eyespots is *endothelial-monocyte activating polypeptide ii*, which functions in inducing the migration of progenitor cells into wound areas with increasing intracellular calcium mobilization in these cells [44]. In addition, *Calcium calmodulin dependent protein kinase* (CAMK) is down-regulated in eyespots. CAMK is a Ca^2+^ dependent kinase [45] which functions as a negative regulator of innate immune responses to cellular damage in the worm [46]. Its down-regulation in eyespots suggests the possible up-regulation of genes involved in wound healing. A *ryanodine receptor like protein* is extremely down-regulated in eyespots. Ryanodine receptor is an intracellular calcium channel [47] that has been implicated in the transmission of slow calcium waves in a variety of tissues. Calcium waves have been observed in *Drosophila* wings discs, especially associated with areas rich with Wingless signals [48], but, so far, this receptor is not known to be expressed in *Drosophila* wing imaginal discs. Wingless and phospho-MAD (a Dpp signal transducer), two other components of the wound repair pathway in *Drosophila* [49], are naturally expressed in *B. anynana* eyespots, shortly after the period of development examined here [26]. These data implicate both calcium signaling and wound healing as candidate pathways involved in the natural process of eyespot development.

Other eyespot-associated genes related to wound healing are the transcription factor *D-Fos*/*Kayak* an effector of both the ERK and JNK signaling pathways [50], and an essential gene for initiation of epithelial repair in *Drosophila* [51]; the *Ras protein,* which induces wound closure in human epidermal cells [52]; and *Chitinase-like protein* which regulates immunity and wound closure in *Drosophila* [53]. Another gene, however, has an opposite expression pattern to that predicted by a simple wound-healing gene regulatory network co-option scenario: *Zinc finger protein* is up-regulated in eyespots. This protein plays a critical role in wound healing as a negative feedback regulator of the Wnt/B-catenin pathway in mammals [54]. Reduced expression of zinc finger protein accelerates cutaneous wound healing in mice [54]. However, this gene is up-regulated in eyespots in our study. This could indicate that if the wound healing regulatory network was co-opted to eyespot development, it is being naturally slowed down in eyespots.

A *Toll-like receptor* and a *Toll-like protein* (also a receptor) are both up-regulated in eyespots. The Toll pathway is known for its ancestral role in activating the innate immune response [55], as well as a derived role in patterning the dorsal ventral axis of insect embryos [56]. Recently, the Toll pathway has also been implicated in wound epidermal closure in *Drosophila* [57]. Both in flies and in worms the immune system is activated by wounding to promote cell survival [40, 43, 55], and signals other than the presence of microbes, such as sterile wounding, can also activate the innate immune response of insects in the absence of an infection. The Toll signaling pathway is also activated in silkworms during immune response [58], and Toll is essential for wound closure in late *Drosophila* embryos [59]. Interestingly, Toll is also expressed in the ectodermal cells that form the leading edge of dorsal closure in *Drosophila* embryos and at intersegmental boundaries [60]. Most recently, the over-expression of constitutively activated Toll was sufficient to induce wound repair genes in the undamaged epidermis of *Drosophila* [57], suggesting that Toll regulates a modular network that may have been co-opted to aid in eyespot origins [61]. Both Toll and its ligand, Spätzle, should be investigated in future regarding their possible role in eyespot development.

### Other new genes and signaling pathways associated with eyespot development

A paralogue of the *yellow* gene, *yellow b*, was found to be down-regulated in eyespots. This gene is downstream of JNK signaling in *Drosophila* and is expressed in the leading edge of the cells involved in dorsal closure [62]. Both JNK and Wg signaling are known to up-regulate *dpp* expression also in the leading edge cells [63] and both Wg and pMad (a Dpp effector gene) transcripts have been visualized in eyespot centers shortly after pupation [26]. This suggests similarities between dorsal closure and eyespot gene regulatory networks in terms of gene identity, but differences at the level of gene regulation (up-regulation of *yellow b* in dorsal closure, down-regulation in eyespots).

This study highlighted a new signaling pathway, the fibroblast growth factor receptor (FGFR) signaling pathway, and confirmed a previously implicated pathway, the bone morphogenetic protein (BMP) pathway, associated with eyespot development at the early stages of pupation, during the focal signaling stage. Two of the differentially regulated genes in eyespots were *kal-1* and *Bmp-binding endothelial regulator protein*. *Kal-1*, which was highly up-regulated in eyespots, codes for anosmin-1 protein, which is involved in the FGFR signaling pathway [64], a pathway with multiple roles in organogenesis, cell differentiation, and wound healing [65]. This gene is also expressed in the embryonic cells of *Drosophila* that will develop into the Keilin’s organs, a larval sensory organ that develops from a sub-set of Dll expressing cells in the thorax, the remaining cells developing into the adult wings and legs [66, 67]. Kal-1 is also expressed in the ectodermal cells that form the leading edge of dorsal closure in *Drosophila* embryos [66]. The second gene, *Bmp-binding endothelial regulator protein,* interacts and inhibits Bone morphogenetic protein (BMP) function [68] but it is down-regulated in eyespot centers, suggesting a role for BMP signaling in promoting eyespot development. Another BMP signaling member, the signal transducer pMAD, was previously visualized in eyespot centers in pupal wings slightly later in development [26].

Our data suggests that local cell division and proliferation may be happening in eyespot-containing tissues as we see the up-regulation of polymerases (*RNA-directed DNA polymerase from mobile element jockey*, and two other “pol-like proteins”) and up-regulation of an apoptosis inhibitor (apoptosis inhibitor 5) associated with eyespots. Scales in the eyespot centers are generally larger than in the neighboring area [69] and these scale cells have increased nuclear volume, suggesting DNA synthesis during the early pupal stages in butterflies [70]. In addition, local cell division in eyespot centers was previously documented to occur during the wandering stages of development [71]. Furthermore, *tp53 regulating kinase*, required to support proliferation and cell growth in *Drosophila* [72], is also up-regulated in eyespots. This suggests that cell division and/or cell growth in eyespot centers may continue from the wandering larval stage up to the early pupal stages.

### New genes with a possible role in eyespot plasticity

Three genes may be playing a yet undiscovered role in eyespot plasticity of dorsal eyespots in *B. anynana* [73]: *alpha-aminoadipic semialdehyde*, *Farnesoic acid O-methyl transferase (FAMeT)* and *Painless*. The top (annotated) up-regulated gene in eyespots is *alpha-aminoadipic semialdehyde* also known in *Drosophila* as lysine ketoglutarate reductase/saccharopine dehydrogenase (LKR/SDH)*,* a co-factor that represses ecdysone mediated transcription of target genes [74]. Ecdysone signaling has been implicated in the regulation of ventral and dorsal eyespot size plasticity at slightly earlier stages of eyespot development (during the wandering stage) [71, 75]. The high expression levels of this repressive co-factor in the early pupal stages may contribute to explain the relative insensitivity of dorsal eyespots to 20E injections performed at this stage of development [74, 76]. The second gene, FAMeT, is an enzyme in the biosynthetic pathway of juvenile hormone (JH) [77]. It is down-regulated in the dorsal eyespots of the wet season form of male butterflies, used in this study. This suggests the possible involvement of JH in either eyespot development, or in the regulation of size and brightness plasticity of dorsal eyespots in males of this species across seasonal forms [73, 78]. Different expression levels of certain isoforms of this enzyme have been previously associated with caste differentiation between queens and workers of the stingless bee [77]. The third gene, *Painless,* is a transient receptor potential channel that acts as a primary harmful heat detector in *Drosophila* [79]. High heat (> 42.6 °C) activates *Painless* expression. It is possible that this gene, if sensitive to lower ambient temperatures in *B. anynana,* may be also involved in regulating eyespot-specific patterns of temperature-mediated plasticity in male eyespots. There is an interesting connection between this gene and calcium signaling described above. Ca^2+^ is known to regulate the function of the *painless* channel in *Drosophila*, and in the absence of Ca^2+^ *painless* fails to respond to heat [79].

### Previously discovered eyespot-associated genes are poorly represented in the transcriptome data

The transcriptome data contained ten of the twelve genes previously associated with eyespot development in *B. anynana,* and other butterflies, at earlier and later stages of eyespot development. These genes include the hox gene *Antennapedia* (*Antp*), the earliest known up-regulated gene in larval wing discs [80], the additional transcription factors *Distal-less* (*Dll*) [24]*, spalt* (*sal*) [26, 31]*, engrailed* (*en*) [25]*, cubitus interruptus* (*ci*) [25]*,* the cell-surface receptors *Notch* (*N*) [81] and *patched* (*ptc*) [25]*,* the nuclear receptor *Ecdysone receptor* (*EcR*) [82–85], the signal transducer *smad* [26], and the segment polarity gene *hedgehog* (*hh*) [25] (Fig. 3). Two other known eyespot-associated genes in *B. anynana*, *Ultrabithorax* (*Ubx*), a hox gene and *wingless* (*wg*), a ligand, were absent from our transcriptome data. The absence of *Ubx* is not unexpected because *Ubx* is expressed only in hindwings in *B. anynana* [86], and in this study tissues were dissected from forewings. The absence of *wg* transcripts might be related to developmental timing. The earliest *wg* mRNA and wg protein expression was identified in the eyespot centers at 10 h and 10.5 h after pupation, respectively [26, 87], and here our dissections were done earlier, between 3 and 6 h. Hedgehog signaling involving *hedgehog* (*hh*), and its receptor *patched* (*ptc*) were previously associated with eyespot development in *Junonia coenia* butterflies [88], but neither *hh* nor *ptc* are expressed in *B. anynana* eyespots [80], and do not seem to function in eyespot development in this species [89].

A surprising finding was that most of the genes previously associated with eyespot development in *B. anynana* were not consistently significantly differentially expressed in tissues with and without eyespots, with the exception of *Antp* and *Dll*. Several possibilities may account for this. The first is that some genes are expressed in cells other than those in the central eyespot region. This non-specificity may have lowered our power to detect significant differential expression across eyespotted and non-eyespotted samples. For instance, *Antp* is expressed strongly and only in eyespot center cells, unlike other genes which are expressed in other cells of the wing sector [80, 86]. For instance, *en* is expressed in all cells of the posterior compartment [80, 88], and *N* is expressed in cells along the midline of each wing sector and also in the wing margin [24, 81, 90].

A second possibility is that since most of the genes previously associated with eyespot development were identified at the protein level, with antibodies (En, Ci, N, Sal, EcR, pSmad, Wg), instead of at the mRNA level (*Dll, hh, ptc*) with in situ hybridizations, it is possible that the protein expression differences are the product of mechanisms of post-transcriptional regulation, rather that transcriptional regulation. Experiments in various organisms including fly and mammalian cells often show that only 40% of the variation in protein levels can be correlated with the abundance of their corresponding mRNAs, and processes involving mRNA or protein degradation can explain this lack of correlation [91].

A third possibility relates to gene expression patterns potentially being very dynamic over time and being absent in eyespots at the time the tissue samples were taken. Most of the genes previously associated with eyespot development were visualized either in the late larval stage and/or around 12-30 h after pupation. No gene has been directly visualized/quantified in eyespots at 3-6 h after pupation because wings are fragile and difficult to handle at this stage. It is possible, thus, that some of the genes only visualized in larval wings (*ci, N*) stop being differentially expressed in young pupal wings, or, alternatively, some of the genes only visualized in older pupal wings (*wg*, *psmad*) start their expression after the time interval examined here. A recent qPCR study of *wg* expression in whole early pupal forewings showed very low expression just after pupation, but detectable levels 6 h later [87].

The transcription factor *Distal-less* (*Dll*) was significantly up-regulated in the eyespotted tissues compared to non-eyespotted tissues. This is an expected result since Dll is expressed in eyespot centers of butterfly wings [24, 26, 90] and has a functional role in eyespot formation [92–94]. Although, two studies demonstrated that this gene is a positive regulator in eyespot development [93, 94], another study claimed a repressive regulatory function for *Dll* in eyespot formation [92]. Our results support the view of a positive regulatory function of *Dll* in eyespot development as *Dll* is expressed in areas with eyespots*.*

### Wing sector specific genes

Several genes were differentially expressed between Cu1 and M3 wing sectors, regardless of presence or absence of eyespots in these sectors. The only annotated transcription factor on our list was *T-box 6*. This gene was over-expressed in M3 wing sectors. A T-box 6 homolog in *Drosophila* is *bifid,* also known as *optomotor-blind* (*omb*) [95, 96]. *omb* is broadly expressed in the middle sectors of the fly wing imaginal disc [97] and is required to induce development of the L5 vein in *Drosophila* [35, 97]. According to the vein nomenclature of Stark et al. [98] the L5 vein in fly wings is homologous to the CuA1 vein (Cu1 vein here), placed between the M3 and Cu1 wing sectors. Hence, our results indicate that the *B. anynana omb* ortholog may have a similar expression pattern and function to *omb* in *Drosophila*. It will be interesting to determine, in future, the exact pattern of expression of this gene on a developing butterfly wing using in situ hybridizations, and the role of this gene, if any, in the control of vein patterning and eyespot development.

### Genes differentially expressed in male and female eyespots

Many genes were differentially expressed between male and female Cu1 dorsal wing sectors with eyespots. Male Cu1 dorsal eyespots display different sizes and patterns of brightness plasticity relative to female eyespots [73, 78], so some of the genes below may be involved in playing a role in the control of this process. The genes that stand out are all expressed at higher levels in females and include *Methoprene-tolerant*, the Juvenile hormone (JH) receptor [99], *JH-inducible protein,* a gene that shows rapid induction in the presence of JH [100], *juvenile hormone binding protein*, a protein carrier of JH to the target cells [101, 102], *bombyxin b-4 precursor*, a mRNA precursor of bombyxin, an insulin-like peptide, which contains many family members [103–106]. The finding that a *bombyxin* mRNA precursor is expressed at higher levels in female eyespots is especially interesting because *bombyxin* family members were previously only known to be expressed in the larval brain, ovaries, fat body, and mid gut of the lepidopteran *Bombyx mori* [104]. Our data suggest a novel expression domain for the B4 family members and their possible role in the regulation of eyespot sexual dimorphism in *B. anynana*.

## Conclusions

The genes associated with eyespot development identified in this work may contain some of the original genes involved in eyespot origins, 90 million years ago [6], as well as *B. anynana* lineage-specific co-options that are not shared across all nymphalid species with eyespots. These lineage-specific co-options are likely to have occurred given the span of time involved since eyespot origins. Genes likely to belong to this latter category include genes regulating eyespot phenotypic plasticity, a likely derived trait within nymphalid butterflies [17], as well as sexual dimorphism in dorsal eyespots. Future comparative transcriptomic experiments, performed across multiple nymphalid species with eyespots, will be required to identify the core set of genes likely associated with eyespot origins and necessary for eyespot development. Currently, our data lends additional support to the hypothesis proposing that eyespots co-opted the wound healing gene regulatory network to aid in their development [26]. The other three hypotheses were not as well supported by the current data because only fewer genes known to be associated with the proposed gene regulatory circuits and networks were also found associated with eyespots. This, however, may simply reflect our incomplete current understanding of these other developmental processes. Future studies should examine genes associated with eyespot development at earlier and later time points, although this may be experimentally challenging. Overall, our study identified many new additional genes associated with eyespot development, eyespot plasticity, and eyespot sexual dimorphism in *B. anynana* that can now be examined across species and also at the functional level.

## Methods

### Animal husbandry

Butterflies were reared in climate controlled chambers at 27 °C on a 12 L: 12D photoperiod. Humidity was kept at 80%. Larvae were fed with corn plants and adults with mashed banana.

### Crossing wild-type individuals with mutant individuals

Wild-type and Spotty lines have been kept separate in the lab for many generations. As a consequence, they may have fixed distinct genetic variants that are not related to the wing pattern mutation. In order to homogenize the genetic background between these lines for the purpose of comparative transcriptomics, female Wt individuals were crossed with Spotty male individuals (Additional file 7: Figure S2). All F1 (first generation) offspring had phenotypes associated with Wt/Spotty heterozygous and displayed small intermediate eyespots in sectors M2 and M3. These F1 individuals were mated with each other to produce a F2 generation. Only wild-type and Spotty homozygous individuals were selected from the F2 generation and these were mated to individuals of the same phenotype to produce two pure-breeding F3 generation cohorts. Female and male wild-type and Spotty pupae from this generation were used for RNA extractions.

### Collecting RNA from wing tissue

Samples from the eyespot centers on the dorsal pupal forewings were used to extract RNA for transcriptome analysis. Small squares of wing tissue of around 0.5 × 0.5 mm, containing the eyespot centers, were dissected between 3 and 6 h after pupation using home-made scalpels with small chips of a razor blade glued to a metal handle (see size of squares in Fig. 1a). Pupation time was estimated with time-lapse photography of pre-pupae, with 30 min intervals between photos, using a Kodak DC290 digital camera. These tissues were collected from two wing sectors, M3 and Cu1 (Fig. 1a). Dissected tissue samples were collected into an eppendorf tube containing RNA*later* reagent (Qiagen) to stabilize the RNA. Around 30 pieces of tissue from the same wing sector and sex were pooled before RNA was extracted with a RNeasy Mini Kit (Qiagen), using a RNase-Free DNase (Qiagen) step. Each of these extractions had concentration between 4 ng/ul and 17 ng/ul in 70 ul of water and was used to make a separate library for RNA sequencing. Three separate libraries were made for each type of tissue to represent biological replicates. RNA concentration was measured with both a Nanodrop 1000 Spectrophotometer (NanoDrop/Thermo Scientific) and Qubit 2.0 Fluorometer (Life Technologies). RNA quality was controlled by agarose gel electrophoresis for RNA degradation and potential contamination, and Agilent 2100 bioanalyser for RNA integrity by AITbiotech. Dissections of M3 and Cu1 squares were performed in random order to avoid any gene expression biases caused by the operation.

### Library preparation and RNA sequencing

RNA library construction was performed with Truseq stranded mRNA kit from Illumina by AITbiotech. mRNA was purified from equal amount of total RNA from each library using oligo(dT) beads to minimize bias for inter-library comparisons. The mRNA was fragmented randomly in fragmentation buffer, followed by cDNA synthesis using random hexamers and reverse transcriptase. After first-strand synthesis, a custom second-strand synthesis buffer (Illumina) was added, with dNTPs, RNase H and *E. coli* polymerase I to generate the second strand and AMPure XP beads were used to purify the cDNA. The final cDNA library was ready after a round of purification, terminal repair, A-tailing, ligation of sequencing adapters, size selection and PCR enrichment (Illumina). Library concentration was first quantified using a Qubit 2.0 fluorometer (Life Technologies), and then diluted to 1 ng/μl before checking insert size on an Agilent 2100 Bioanalyzer and quantifying to greater accuracy by quantitative PCR (Kapa Biosystems) (library activity >2 nM). Libraries were loaded into a Illumina Hiseq 2000 (next-generation sequencing machine) according to activity determined from qPCR and expected data volume of the instrument by AITbiotech. One lane was used to sequence a total 15 libraries from wild-type and Spotty lines. After sequencing, adaptors were removed from sequences and initial quality check reports for raw reads were provided by AITbiotech.

### Transcriptome assembly

We first re-constructed transcripts from the short reads using a “de novo assembly” approach [107]. Around 400 million paired-end reads were used for de novo assembly. Both Trinity and CLC genomics workbench were used for the transcriptome assembly using different assembly and trimming parameters. The quality of the various assembled transcriptomes was assessed according to average transcript’s length, the number of transcripts, and the coverage of aligned transcripts against the protein database of another lepidopteran, *Bombyx mori*. Transcriptomes were deemed to be of higher quality based on longest N50 value, fewer number of transcripts, and highest full-length coverage of all known proteins of *Bombyx mori.* According to these criteria, the best transcriptome was built using Trinity software using all 15 libraries and the following parameters: Quality trimming was performed with trimmomatic [108], which is integrated into Trinity software. Reads were trimmed at the start and the end of a read, if the threshold quality was below 5, and reads less than 30 bp were removed prior to the assembly (e.g., LEADING:5, TRAILING:5, MINLEN:30). After assembly, transcripts under 200 bp were removed from the final transcriptome.

### Identifying differentially expressed genes

Differentially expressed genes were identified with the downstream applications of the Trinity software package [109]. These methods use RSEM software for estimating transcript abundance [110] and EdgeR Bioconductor package for identifying differentially expressed genes across samples after sample normalization for scaling library size using a weighted trimmed mean of the log expression ratios (trimmed mean of M values (TMM)) [111, 112]. A *p*-value of 0.05 was chosen as the threshold to identify statistically significant up and down-regulated genes between libraries.

### Gene annotations and enrichment analysis

Differentially expressed genes were blasted against a non-redundant protein database with blastx using an E-value <1e-3 (E-value (expected value) shows the number of significant alignments expected to occur by chance) and were annotated for gene functions using Blast2GO software [113]. Afterwards, these transcripts were annotated using Blast2GO and were added into the list of previously annotated genes for further analyses. Gene ontology terms were found using non-redundant protein database in Blast2GO (E-value <1e-3). A test for functional enrichment of particular gene ontologies among the 186 eyespot associated genes was performed using Fisher’s exact test and the *Drosophila* database as a reference on Blast2GO PRO plugin for CLC genomic workbench.

### Co-option of the gene regulatory networks

All of the annotated eyespot associated genes were searched for their functions, related to the proposed co-opted gene regulatory network using keywords; “wound healing”, “limb development”, “anterior-posterior axis”, “segment polarity” and “wing margin” in the Google Scholar search engine.

### Candidate gene approach

First, the sequences of the previously identified candidate eyespot genes were downloaded from the nucleotide database of NCBI. Second, these sequences were aligned to the transcriptome with a Blastn command in BLAST+ (E-value <1e-20) and the longest transcript of the corresponding gene was selected from the Blastn results. Later, a *p*-value and a logFC value of the transcripts of the candidate eyespot genes were obtained from the M3 wing sectors comparison between wild-type and Spotty tissues.

## Additional files

Additional file 1: Figure S1. Hierarchical clustering of similarities in gene expression across the fifteen libraries. Color bars at the top of the figure represent libraries from the following tissue samples: Pink (female wild-type Cu1), blue (wild-type Cu1), green (wild-type M3), yellow (spotty Cu1), red (Spotty M3). Rows represent significantly differentially expressed genes across libraries clustered by their similar expression patterns. Color key indicates up (red) and down (green) regulated genes according to their log fold changes. (TIF 10.2 mb)

Additional file 2: Table S1. *P*-values and logFC (log2-fold change) of differentially expressed genes associated with eyespot development across all three comparisons. Negative logFC values represents up-regulated genes in eyespots, because the values from tissues without eyespots were divided by those tissues with eyespots. (XLSX 152 kb)

Additional file 3: Table S14. Blast2GO annotations for up-regulated genes associated with eyespots. **Table S15.** Blast2GO annotations for down-regulated genes associated with eyespots. **Table S16.** Blast2GO annotations for up-regulated sector-specific transcripts in M3 versus Cu1 wing sector. **Table S17.** Blast2GO annotations for down-regulated sector-specific transcripts in M3 versus Cu1 wing sector. **Table S18.** Blast2GO annotations for up-regulated genes in female versus male eyespots. **Table S19.** Blast2GO annotations for down-regulated genes in female versus male eyespots. Each table is in a different tab. (XLSX 82 kb)

Additional file 4: Table S3. Total transcript raw read counts and TMM values of genes previously associated with butterfly eyespot development across the four types of tissue sample (each with three replicates) used to identify the genes associated with eyespot development (see Fig. 1c). Multiple rows for each gene represent different non-overlapping transcripts obtained for the same gene. (XLSX 45 kb)

Additional file 5: Table S2. Eyespot associated genes in the previously proposed co-opted gene regulatory networks. (DOCX 122 kb)

Additional file 6: Table S4. Up-regulated genes in M3 wing sector in Spotty versus M3 wing sector in wild-type. **Table S5.** Down-regulated genes in M3 wing sector in Spotty versus M3 wing sector in wild-type. **Table S6.** Up-regulated genes in Cu1 wing sector in wild-type versus M3 wing sector in wild-type. **Table S7.** Down-regulated genes in Cu1 wing sector in wild-type versus M3 wing sector in wild-type. **Table S8.** Up-regulated genes in Cu1 wing sector in Spotty versus M3 wing sector in wild-type. **Table S9.** Down-regulated genes in Cu1 wing sector in Spotty versus M3 wing sector in wild-type. **Table S10.** Up-regulated genes in M3 wing sector in Spotty versus Cu1 wing sector in Spotty. **Table S11.** Down-regulated genes in M3 wing sector in Spotty versus Cu1 wing sector in Spotty. **Table S12.** Up-regulated genes in Cu1 wing sector in male (wild-type) versus Cu1 wing sector in female (wild-type). **Table S13.** Down-regulated genes in Cu1 wing sector in male (wild-type) versus Cu1 wing sector in female (wild-type). Each table is in a different tab. (XLSX 790 kb)

Additional file 7: Figure S2. Crosses between wild-type and Spotty butterflies. Female Wt individuals were crossed with Spotty male individuals. All F1 (first generation) offspring had phenotypes associated with wt/Spotty heterozygous and displayed small intermediate eyespots in sectors M2 and M3. These F1 individuals were mated with each other to produce a F2 generation. Only wild-type and Spotty homozygous individuals were selected from the F2 generation and these were mated to individuals of the same phenotype to produce two pure-breeding F3 generation cohorts. Female and male wild-type and Spotty pupae from this generation were used for RNA extractions. (TIFF 860 kb)

## Abbreviations

BMP: Bone morphogenetic protein
ERK: Extracellular signal–regulated kinase
FDR: False discovery rate
FGF: Fibroblast growth factor
FGFR: Fibroblast growth factor receptor
GO: Gene ontology
JNK: Jun N-terminal kinase
logFC: Logarithmic fold change
Wt: Wild-type
