# Supplementary figures and images for: Wound healing, calcium signaling, and other novel pathways are associated with the formation of butterfly eyespots
Source: BMC Genomics. 2017 Oct 16;18:788. doi: 10.1186/s12864-017-4175-7 (PMC5644175; doi:10.1186/s12864-017-4175-7)

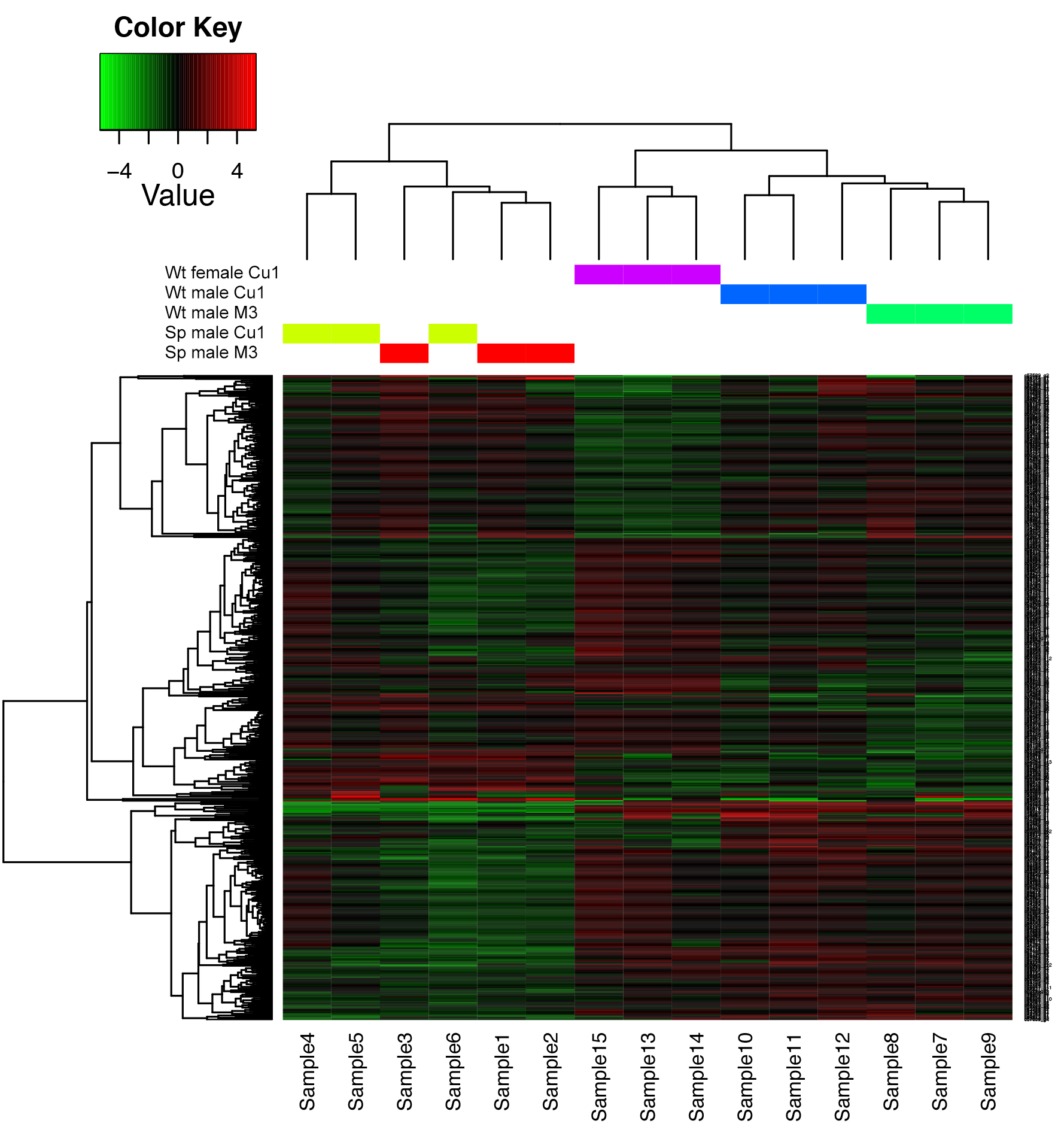

Supplement: Supplementary file 1 — Hierarchical clustering of similarities in gene expression across the fifteen libraries. Color bars at the top of the figure represent libraries from the following tissue samples: Pink (female wild-type Cu1), blue (wild-type Cu1), green (wild-type M3), yellow (spotty Cu1), red (Spotty M3). Rows represent significantly differentially expressed genes across libraries clustered by their similar expression patterns. Color key indicates up (red) and down (green) regulated genes according to their log fold changes. (TIF 10.2 mb) [file 12864_2017_4175_MOESM1_ESM.tif]

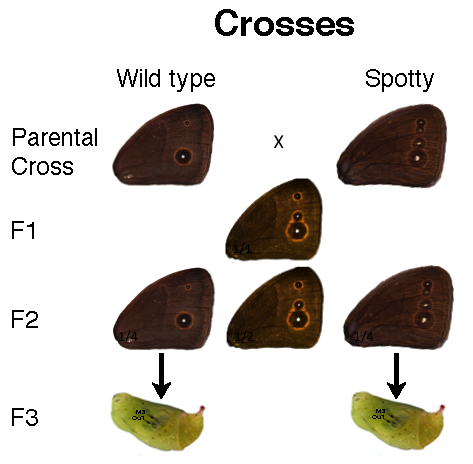

Supplement: Supplementary file 7 — Crosses between wild-type and Spotty butterflies. Female Wt individuals were crossed with Spotty male individuals. All F1 (first generation) offspring had phenotypes associated with wt/Spotty heterozygous and displayed small intermediate eyespots in sectors M2 and M3. These F1 individuals were mated with each other to produce a F2 generation. Only wild-type and Spotty homozygous individuals were selected from the F2 generation and these were mated to individuals of the same phenotype to produce two pure-breeding F3 generation cohorts. Female and male wild-type and Spotty pupae from this generation were used for RNA extractions. (TIFF 860 kb) [file 12864_2017_4175_MOESM7_ESM.tif]
