# Supplementary material for: Wound healing, calcium signaling, and other novel pathways are associated with the formation of butterfly eyespots
Source: BMC Genomics. 2017 Oct 16;18:788. doi: 10.1186/s12864-017-4175-7 (PMC5644175; doi:10.1186/s12864-017-4175-7)
Supplement: Supplementary file 5 — Eyespot associated genes in the previously proposed co-opted gene regulatory networks. (DOCX 122 kb) [file 12864_2017_4175_MOESM5_ESM.docx]

**S2 Table. Eyespot associated genes in the previously proposed co-opted gene regulatory networks**

| Regulation | SeqName | Description | Wound healing | Limb development | Anterior-posterior axis | Wing margin development |
| --- | --- | --- | --- | --- | --- | --- |
| Up | c59701_g2 | pol-like protein | - | - | - | - |
|  | c37759_g2 | alpha-aminoadipic semialdehyde synthase, mitochondrial/ lysine ketoglutarate reductase/saccharopine dehydrogenase (LKR/SDH) | - | - | - | - |
|  | c51288_g1 | ubiquitin carboxyl-terminal hydrolase isozyme L5 | ✔ | - | - | - |
|  | c57271_g5 | rna-directed dna polymerase from mobile element jockey- partial | - | - | - | - |
|  | c55817_g1 | kish | - | - | - | - |
|  | c56277_g1 | chitinase-like protein | ✔ | - | - | ✔ |
|  | c53127_g1 | cuticle 3-like | - | - | - | - |
|  | c59133_g1 | kal-1 protein isoform x2 | ✔ | ✔ | - | ✔ |
|  | c58938_g2 | Adenylyltransferase and sulfurtransferase MOCS3 | - | - | - | - |
|  | c58691_g6 | fatty acid synthase | - | - | ✔ | - |
|  | c56057_g2 | zinc transporter 1 | ✔ | - | - | - |
|  | c51626_g1 | toll-like receptor 13 | ✔ | - | - | - |
|  | c64109_g1 | Retrovirus-related Pol poly from transposon opus | - | - | - | - |
|  | c47739_g1 | lysosomal alpha-mannosidase isoform X1 | - | - | - | - |
|  | c62711_g2 | fatty acyl- reductase 1-like | - | - | - | - |
|  | c54013_g2 | elongation of very long chain fatty acids protein aael008004-like | - | - | - | - |
|  | c59504_g1 | protein toll-like | ✔ | - | - | - |
|  | c48574_g1 | hypothetical protein KGM_03542 | - | - | - | - |
|  | c53839_g1 | apoptosis inhibitor 5 | - | - | - | - |
|  | c49947_g1 | U11 U12 small nuclear ribonucleo 48 kDa -like | - | - | - | - |
|  | c56619_g4 | sodium potassium-transporting atpase subunit beta-2 | - | - | - | - |
|  | c62730_g2 | transient receptor potential cation channel protein painless | - | - | - | ✔ |
|  | c49643_g1 | circadian clock-controlled isoform x1 | - | - | - | - |
|  | c55365_g1 | cysteine-rich motor neuron 1 protein | ✔ | - | - | - |
|  | c64590_g1 | protein ergic-53 | - | - | - | - |
|  | c63228_g4 | glucose dehydrogenase | - | - | ✔ | - |
|  | c56040_g1 | roundabout 1 | - | - | - | - |
|  | c58348_g1 | pol-like protein | - | - | - | - |
|  | c14558_g1 | Keratin-associated protein 19-2-like | ✔ | - | - | - |
|  | c60618_g1 | cutilin-1 precursor | - | - | - | - |
|  | c58131_g1 | omega-scoloptoxin-ssm1a isoform x1 | - | - | - | - |
|  | c54010_g1 | signal recognition particle 19 kda protein | - | - | - | - |
|  | c57413_g1 | hypothetical protein KGM_15760 | - | - | - | - |
|  | c52331_g1 | nad-dependent protein deacetylase sirt6 | ✔ | - | - | - |
|  | c47756_g2 | chemosensory protein 11b | - | - | ✔ | - |
|  | c55395_g1 | PREDICTED: uncharacterized protein LOC106137651 isoform X2 | - | - | - | - |
|  | c56453_g2 | serine threonine-protein kinase prp4 homolog isoform x1 | - | - | - | - |
|  | c38859_g1 | signal peptidase complex subunit 1 | - | - | - | - |
|  | c59733_g1 | trna (guanine-n -)-methyltransferase | - | - | - | - |
|  | c55214_g1 | probable arginine--trna mitochondrial | - | - | - | - |
|  | c53186_g3 | transcription elongation factor 1 homolog | - | - | - | - |
|  | c60403_g1 | upf0746 protein ddb_g0281095-like isoform x1 | - | - | - | - |
|  | c53596_g1 | alpha-tocopherol transfer | - | - | - | - |
|  | c58621_g3 | dynein light chain roadblock-type 2-like | - | - | - | - |
|  | c55791_g1 | gpi-anchored wall transfer protein 1 | - | - | - | - |
|  | c58300_g3 | serine--trna mitochondrial | - | - | - | - |
|  | c55878_g3 | zinc finger protein 830 | ✔ | - | ✔ | - |
|  | c50373_g1 | 39s ribosomal protein mitochondrial | - | - | - | - |
|  | c56899_g1 | protein tssc4 | - | - | - | - |
|  | c56760_g1 | methyltransferase nsun6 | - | - | - | - |
|  | c59338_g1 | ribosome-recycling mitochondrial | - | - | - | - |
|  | c57588_g1 | PREDICTED: uncharacterized protein LOC106140627 isoform X2 | - | - | - | - |
|  | c57103_g1 | unconventional prefoldin rpb5 interactor | - | - | - | - |
|  | c21133_g1 | rna-binding protein nob1 | ✔ | - | - | - |
|  | c54208_g1 | eukaryotic translation initiation factor 3 subunit k | - | - | - | - |
|  | c52002_g1 | endothelial-monocyte activating polypeptide ii | ✔ | - | - | - |
|  | c49068_g1 | peptidyl-prolyl cis-trans isomerase-like 3 | - | - | - | - |
|  | c52387_g2 | signal peptidase complex subunit 3 | - | - | - | - |
|  | c55727_g1 | tp53-regulating kinase | ✔ | - | - | - |
|  | c58476_g2 | DnaJ homolog subfamily C member 17 | - | - | - | - |
|  | c48280_g1 | mki67 fha domain-interacting nucleolar phospho | - | - | - | - |
|  | c39919_g1 | mesencephalic astrocyte-derived neurotrophic factor homolog | - | - | - | - |
|  | c51381_g3 | coiled-coil domain-containing protein 58 | - | - | - | - |
|  | c59405_g1 | srr1-like protein | - | - | - | - |
|  | c61066_g1 | protein aatf | - | - | - | - |
|  | c54507_g1 | thap domain-containing protein 9 | - | - | - | - |
|  | c61634_g1 | Transmembrane and coiled-coil domain-containing 7 | - | - | - | - |
|  | c27864_g1 | surfeit locus protein 6 homolog | - | - | - | - |
|  | c62633_g1 | eukaryotic translation elongation factor 1 epsilon-1 | - | - | - | - |
|  | c44449_g1 | zinc finger ccch domain-containing protein 3 | - | - | - | - |
|  | c86274_g1 | protein ecdysoneless homolog | - | - | - | - |
|  | c57902_g1 | RNA pseudouridylate synthase domain-containing 2-like isoform X1 | - | - | - | - |
|  | c60700_g3 | rrna-processing protein fcf1 homolog | - | - | - | - |
|  | c63127_g1 | cellular retinaldehyde-binding protein | - | - | - | - |
|  | c56989_g1 | nitric oxide synthase-interacting protein homolog | - | - | - | - |
| Down | c61787_g1 | uncharacterized loc106106657 precursor | - | - | - | - |
|  | c49995_g1 | transcription factor kayak isoform x2 | ✔ | - | - | ✔ |
|  | c60243_g1 | tetratricopeptide repeat protein 17 | - | - | - | - |
|  | c63656_g2 | syntaxin-1A isoform X5 | - | - | - | - |
|  | c63830_g1 | synaptonemal complex | - | - | - | - |
|  | c60221_g1 | Sortilin-related receptor | ✔ | - | - | - |
|  | c54631_g1 | ryanodine receptor-like protein | - | - | - | - |
|  | c51050_g1 | rna-directed dna polymerase from mobile element jockey-like | - | - | - | - |
|  | c3841_g1 | ras-like protein 3 | ✔ | - | - | - |
|  | c58060_g1 | ras gtpase-activating protein 1 | - | - | - | - |
|  | c58269_g1 | protein yellow | ✔ | - | - | - |
|  | c38585_g1 | protein diaphanous | - | - | - | - |
|  | c59543_g1 | probable multidrug resistance-associated protein lethal 03659 | - | - | - | - |
|  | c42554_g1 | PREDICTED: uncharacterized protein LOC106108988 | - | - | - | - |
|  | c55596_g2 | phosphatidylinositol -bisphosphate 3-kinase catalytic subunit delta isoform | - | - | - | - |
|  | c46236_g1 | PH and SEC7 domain-containing 3 isoform X1 | - | - | - | - |
|  | c59815_g1 | neutral ceramidase | - | - | - | - |
|  | c38400_g1 | larval cuticle protein lcp-22-like precursor | - | - | - | - |
|  | c49404_g1 | large proline-rich BAG6 | - | - | - | - |
|  | c25283_g1 | kinase suppressor of ras 2-like protein | - | - | - | - |
|  | c53884_g1 | hypothetical protein RR46_02196 | - | - | - | - |
|  | c77406_g1 | hypothetical protein RR46_01763 | - | - | - | - |
|  | c55061_g1 | hypothetical protein KGM_11484 | - | - | - | - |
|  | c54992_g1 | glycosyltransferase 25 family member | - | - | - | - |
|  | c48616_g1 | fatty-acid amide hydrolase 2-like | - | - | - | - |
|  | c53282_g2 | farnesoic acid o-methyltransferase-like isoform 1 protein | - | - | - | - |
|  | c59651_g1 | DNA-dependent kinase subunit | - | - | - | - |
|  | c50606_g1 | denn domain-containing protein 4c isoform x1 | - | - | - | - |
|  | c55818_g1 | cytoplasmic dynein 2 heavy chain 1 | - | - | - | - |
|  | c9511_g1 | cereblon | - | ✔ | - | - |
|  | c29885_g1 | cd63 antigen | - | - | - | - |
|  | c44374_g1 | calcium calmodulin-dependent protein kinase kinase 2 isoform x2 | ✔ | - | - | - |
|  | c53148_g1 | bmp-binding endothelial regulator protein | - | - | - | - |
|  | c61550_g4 | beta-secretase 1-like | - | - | - | - |
|  | c61396_g1 | antennal esterase cxe5 | - | - | - | - |
|  | c55855_g1 | amp deaminase 2 isoform x5 | - | - | - | - |
|  | c41550_g1 | acid alpha-glucosidase | - | - | - | - |
|  | c14112_g1 | Ac1147 partial | - | - | - | - |
|  | c50808_g1 | abc atp-binding protein | - | ✔ | - | - |
|  |  |  | 18 | 3 | 4 | 4 |
